# Size Dependence of the Band Gap of Core–Shell Tantalum and Tantalum Oxide (V) Nanoclusters

**DOI:** 10.3390/nano15010014

**Published:** 2024-12-26

**Authors:** Valentin A. Shilov, Petr V. Borisyuk, Diana V. Bortko, Smagul Karazhanov, Yuri Y. Lebedinskii, Oleg S. Vasilyev

**Affiliations:** 1 Department No. 78 Physical and Technical Problems of Metrology, National Research Nuclear University MEPhI (Moscow Engineering Physics Institute), 115409 Moscow, Russia; valentineshilov@yandex.com (V.A.S.); pvborisyuk@mephi.ru (P.V.B.); diana.bortko@gmail.com (D.V.B.); yylebedinskij@mephi.ru (Y.Y.L.); osvasilyev@mephi.ru (O.S.V.); 2 Department for Solar Energy Materials and Technologies, Institute for Energy Technology, 2027 Kjeller, Norway; 3 Center of Shared Facilities in Nanotechnology, Moscow Institute of Physics and Technology (MIPT), 141700 Dolgoprudny, Russia

**Keywords:** nanoclusters, nanoparticles, tantalum, tantalum oxide, band gap, quadrupole mass filter, REELS, XPS

## Abstract

Monodisperse films of spherical tantalum oxide (V) nanoclusters and spherical tantalum nanoclusters with a tantalum oxide shell with diameters of 1.4–8 nm were obtained by magnetron sputtering. The size of the deposited nanoclusters was controlled using a quadrupole mass filter. The chemical composition was certified using the XPS method. Using the Reflected Electron Energy Loss Spectroscopy (REELS), the dependence of the band gap width on the nanocluster size was determined. It was found that starting from a certain nanocluster size, the band gap width increases as the nanocluster size decreases. Based on experimental data and a theoretical model, the effective mass of electrons dependence as a function of nanocluster size was obtained.

## 1. Introduction

Nanoclusters, being an intermediate object between individual atoms and bulk matter, allow us to study changes in the properties of substances with a decrease in the number of atoms. At the same time, these properties can change significantly depending on factors such as the size of the nanoparticles, chemical composition, shape and morphology. One of these effects is the change in the density of electron states of a nanocluster near the Fermi energy depending on their size, which can be detected by scanning tunnel spectroscopy. The change in density of electron states leads to a change in the permittivity of the material, which leads to a change in the optical and electronic properties of the material. At the same time, in a material consisting of metal nanoclusters with small diameters, a band gap can be observed [1]. In addition to copper, nanoparticles of refractory metals, such as tantalum, are also an interesting subject for research. Tantalum is widely used in electronics since tantalum (V) oxide has a high permittivity [2], enabling the creation of capacitors with high specific capacitance on its basis and making it a promising material for selective optical coatings [3]. Furthermore, tantalum is a refractory metal, which allows the use of materials based on it under high temperatures (up to 1200 °C). Due to the oxidation of tantalum nanoclusters in the atmosphere and upon contact with oxygen, forming Ta_2_O_5_, it is difficult to use wet and dry chemistry methods to obtain them, such as sol–gel, colloidal reactors, and hydrothermal synthesis.

However, it is possible to use methods in high vacuum conditions, such as pulsed laser deposition (PLD) and magnetron sputtering. The PLD method allows nanoparticle films in vacuum conditions to be obtained. For example, it was previously demonstrated that it is possible to obtain nanoclusters with rough fractal surfaces and non-spherical shapes [4,5] as well as spherical nanoclusters [6,7]. For instance, in [7], films of nanoparticles with a diameter of 69 nm and standard deviation of 26 nm were obtained. Unfortunately, it is difficult to obtain multilayer films consisting of monodisperse nanoclusters using the PLD method without using any size-filtering technique.

To solve these problems in the present article, we used the combination of magnetron sputtering and quadrupole mass-filtering methods to obtain monodisperse spherical Ta_2_O_5_ nanoclusters and core–shell nanoclusters of tantalum and its oxides. The method for obtaining nanoclusters is similar to that described in [8,9,10], with the exception that oxidation occurs directly during the deposition. Previously, for individual spherical tantalum metal nanoclusters of different sizes, a change in the differential current–voltage characteristics as a function of the diameter, measured by scanning tunneling spectroscopy, was discovered. This indicates the dependence of the density of electronic states on the nanocluster size [11]. Also, for thin films of tantalum oxide nanoclusters, such effects as a shift in the binding energy as a function of the size of the nanoclusters [9] and how the optical transmittance of nanocluster films depends on the size of the nanoclusters [12] were discovered.

In addition to metallic tantalum, tantalum oxides are also of great interest to researchers. Since Ta_2_O_5_ is a wide-band-gap dielectric [13], it is possible to create nanoscale systems on its basis by varying the parameters, which makes it possible to change the band gap within fairly wide limits. For example, this was previously demonstrated using a system consisting of thin layers of Ta_2_O_5_ and SiO_2_ [3]. In addition to one-dimensional [14] and two-dimensional [15] nanosystems, quantum dots, which are spherical nanoclusters, also have special quantum properties due to the quantum confinement phenomenon [16]. It was previously found that the band gap and valence band offset could depend on the core–shell nanocluster diameter [8]. Unfortunately, in [8], nanoclusters were oxidized in the atmosphere, which did not make it possible to guarantee the purity of the samples and the constant thickness of the shell [17]. In addition, individual nanoclusters in porous films could oxidize in the atmosphere differently [18] depending on their position and surroundings.

In this work, new materials based on monodisperse core–shell nanoclusters with a core consisting of tantalum metal and a tantalum oxide shell, as well as Ta_2_O_5_ nanoclusters, are studied. All clusters were oxidized directly during deposition, which ensured uniform oxidation of nanoclusters throughout the entire nanocluster film, regardless of their depth. The samples were analyzed in situ using X-ray photoelectron spectroscopy (XPS) and reflected electron energy loss spectroscopy (REELS). The band gap dependence on the size of the nanoclusters was found and studied. Adjusting the band gap width allows for the optical properties of the material [10,14] to be tuned, which is important for creating selective optical coatings, nonlinear optical materials [19] and photodetectors [20].

## 2. Materials and Methods

**Figure 1:**
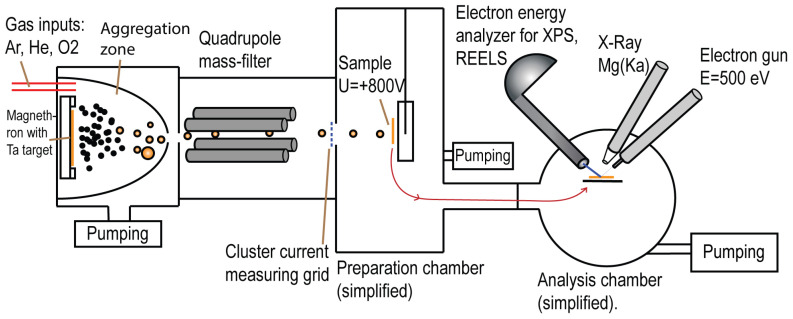
Experimental setup diagram.

To obtain spherical Ta_2_O_5_ nanoclusters and core–shell nanoclusters of tantalum and its oxides, the magnetron sputtering method is used using a Nanogen-50 cluster source with MesoQ quadrupole mass filter (Mantis Deposition Ltd., Thame, UK). Using a dc magnetron in the aggregation zone in an atmosphere of buffer gas—argon (pressure P = 10^−4^ mbar − 10^−3^ mbar), the tantalum target (99.95% purity tantalum) is sputtered, and aggregation of tantalum into nanoclusters occurs. Typical gas flows into the chamber are Ar: 15–50 sccm, He: 0–15 sccm, and O_2_: 0.2–5.0 sccm. Then, the gas flows through a narrow nozzle with a diameter of d = 3 mm and enters a quadrupole mass filter, which filters nanoclusters by mass. The mass-filter consists of four parallel rods, with opposing rods having the same electrical potential. A combination of alternating voltage (AC) and a direct current (DC) voltage is applied to these rods: ($U+Vcos\left(\omega t\right)$ on the first pair and $-(U+Vcos\left(\omega t\right))$ on the second pair of rods. The combined effect of these voltages creates regions of stability and instability for the nanoclusters passing the mass filter, as described by the Mathieu equations. In the coordinates a(q), the region of solutions of the equations of motion for which nanoclusters pass through the mass filter has the shape close to the triangle, where $a=\frac{8\mathrm{eU}}{mr_{0}^{2}\omega^{2}}$, $q=\frac{4\mathrm{eV}}{mr_{0}^{2}\omega^{2}}$, $r_{0}$ is the distance between the rods and the center of the quadrupole. By choosing appropriate a and q values, the quadrupole can be tuned to allow ions with specific m/z ratios to pass through, effectively filtering the ions based on their mass. In these experiments, U/V=a/q=0.04, which made it possible to obtain a flow of monodisperse clusters with a size deviation of Δd/d < 0.03. As all clusters have a charge equal to the charge of the electron (e), adjusting the frequency (ω) of AC voltage leads to a change in the size of the transmitted nanoclusters. The addition of oxygen allows for Ta and Ta_2_O_5_ to be obtained, as well as core–shell nanoclusters with a shell of Ta_2_O_5_ and non-stoichiometric oxides TaO_2_ and Ta_2_O_3_. After a quadrupole mass filter, nanoclusters were deposited on a silicon substrate under a potential of +800 V located in the adjacent chamber. The potential value was chosen to be sufficient to focus the flow of nanoclusters onto the sample and achieve good adhesion but not too large to affect the shape of the nanoclusters upon collision with the substrate. In addition, it prevents positively charged buffer gas ions from reaching the sample, which could etch away the already deposited clusters. The scheme of the experiment is shown in Figure 1.

**Figure 2:**
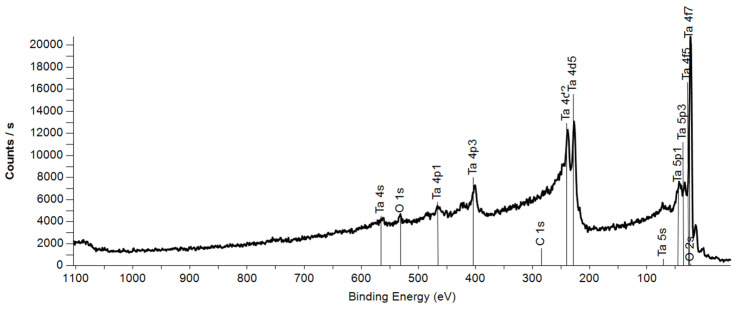
Survey XPS spectrum of core–shell tantalum nanoclusters with diameter d = 3 nm.

This method was used to obtain samples of nanocluster films on silicon of two types: core–shell nanoclusters Ta+TaOx (samples with average nanocluster diameters (d) of 1.5, 2.0, 4.5, 3.9, 8.1 nm) and Ta_2_O_5_ nanoclusters (samples with average nanocluster diameters of 1.5, 1.6, 2, 3.8 nm). During the deposition process, the cluster current (I) was monitored and recorded, and the value of $k=\int_{0}^{t}I\left(t\right)d^{3}dt$ calculated continuously. The relation between the k and deposited mass was previously calibrated using quartz crystal microbalance (QCM). This made it possible to obtain the same film thickness (25 nm) for different nanocluster samples, stopping deposition when a certain value of k was reached. The X-ray photoelectron spectroscopy (XPS) method was used to certify the chemical composition of the obtained nanocluster film. The XPS spectrometer, as well as the electron gun for the Reflected Electron Energy Loss Spectroscopy (REELS) method, are located in the adjacent chamber of the same vacuum facility, which allows in situ studies of the obtained samples without exposure to the atmosphere, which allows for a high frequency of samples, as well as avoiding complete oxidation of core–shell nanoclusters. A typical wide XPS spectrum of a sample of core–shell tantalum nanoclusters on silicon is shown in Figure 2. There are no silicon peaks in the spectrum, which indicates a sufficiently large thickness of the nanocluster film. The absence of carbon peaks indicates the absence of organic contaminants in the sample.

To check the homogeneity of the chemical composition of the obtained nanocluster film, the angular dependences of the XPS were measured and analyzed using the same technique as in [21]. The processing results did not reveal any difference in the intensity ratio of the peak areas of oxygen (O1s) and tantalum (Ta 4f 7/2) at different angles between the sample and the direction of the energy analyzer (all ratios coincided within the experimental error), which indicates the homogeneity of the chemical composition of the obtained nanocluster film in the direction normal to the plane of the sample. In addition, the homogeneity of pure tantalum nanocluster films deposited in this experimental facility was previously assessed using scanning electron microscopy [9].

**Figure 3:**
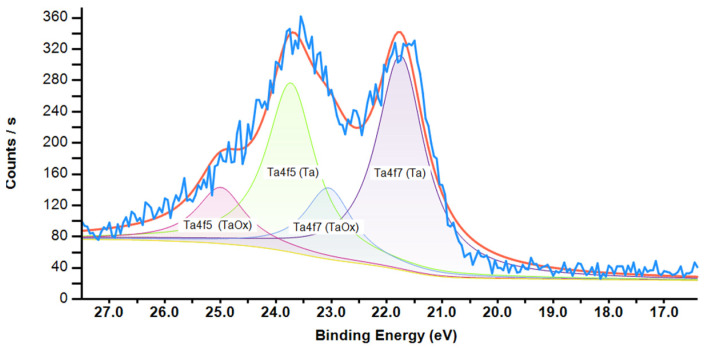
XPS spectrum of Ta+TaO_x_ nanoclusters, Ta4f doublet.

**Figure 4:**
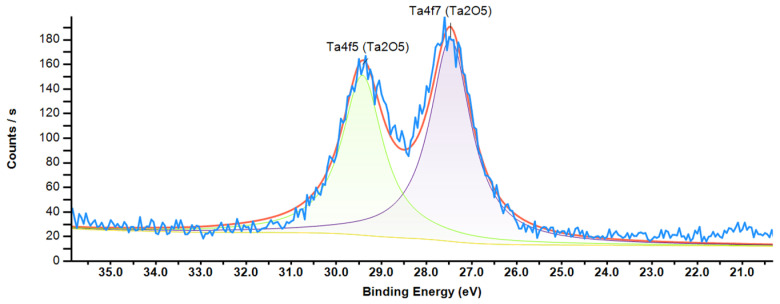
XPS spectrum of Ta_2_O_5_ nanoclusters, Ta4f doublet.

To determine the stoichiometry of nanoclusters and the thickness of the core–shell of Ta+TaOx nanoclusters, the peaks of tantalum and oxygen were recorded in high resolution. A typical region of the Ta4f doublet of the XPS spectrum of core–shell nanoclusters and Ta_2_O_5_ nanoclusters is shown in Figure 3 and Figure 4.

**Figure 5:**
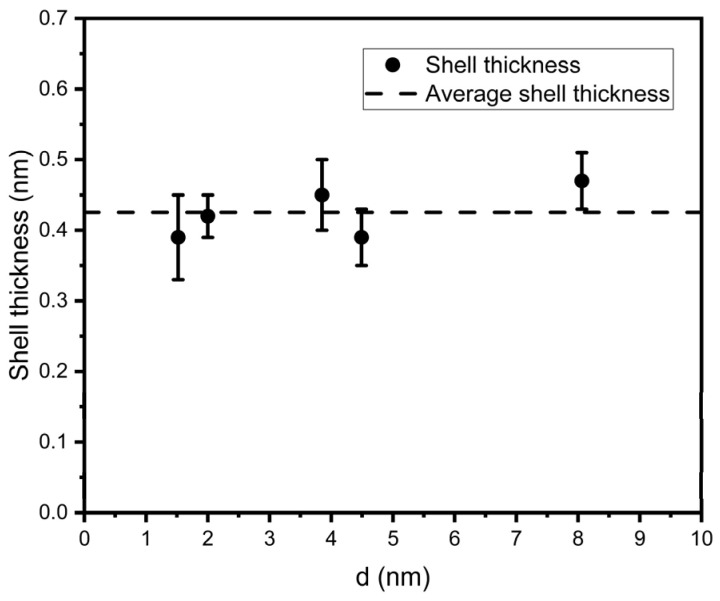
The dependence of TaO_x_ shell thickness of Ta+TaO_x_ core–shell nanocluster on nanocluster diameter.

Using these data, the thickness of the oxide layer for core–shell nanoclusters was calculated from the ratios of contributions from the Ta 4f peaks using the well-known technique [22]; the results of the calculation are shown in Figure 5.

Since, within the limits of experimental error, the shell thickness does not depend on the nanocluster diameter (the maximum change in shell thickness is smaller than the Ta lattice parameter), we will further designate samples only on the basis of the nanocluster diameter.

## 3. Results

**Figure 6:**
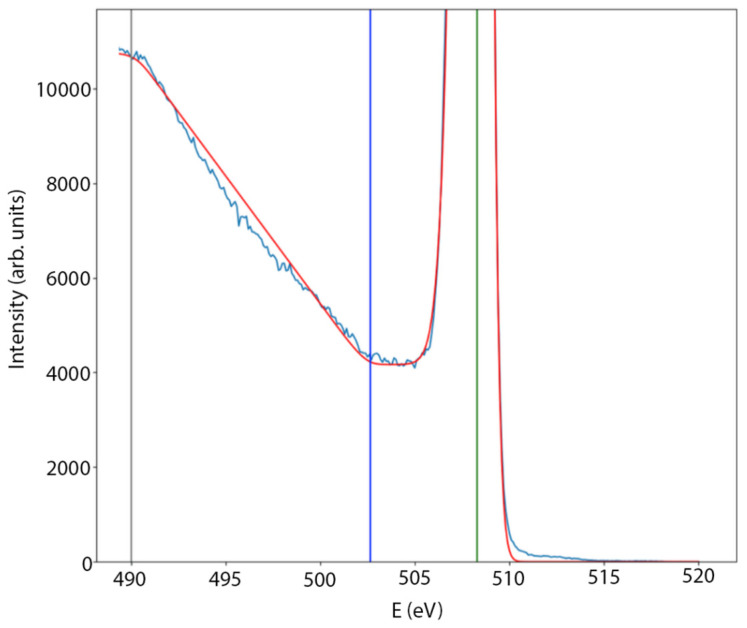
The blue line represents the REELS spectrum of a core–shell nanocluster sample with a nanocluster diameter of d = 4.5 nm. The red line represents the approximation of REELS data according to the model. The green vertical line represents the point of maximum elastic peak. The grey vertical line represents the model approximation region border. The blue vertical line represents the band gap region start.

The band structure analysis was performed using the REELS method. For each experiment, an electron beam energy of 500 eV was used. The typical REELS spectra of a core–shell nanocluster sample with a nanocluster diameter of d = 4.5 nm are shown in Figure 6.

Based on these data, the band gap width was determined for all samples of nanocluster films. For a more accurate determination of the band gap width, the REELS spectra near the band gap and elastic peak were approximated by a model function. The model consists of the convolution of the step function ($I_{mod}(E))$ with a model hardware function ($S_{d}(E))$, which is a combination of the Lorentz and Gaussian functions: $I_{res}(E)=I_{mod}(E)*S_{d}(E)$ where the hardware function is defined as follows:

$$S_{d}\left(E\right)=\left\{\begin{array}{c} \frac{1}{\left(1+M\frac{{\left(E-E_{0}\right)}^{2}}{\beta^{2}})exp(\frac{\left(1-M\right)\ln\left(2\right)\;{\left(E-E_{0}\right)}^{2}\;}{\beta^{2}}\right)}\;,\;E>E_{0}\\ \frac{1}{\left(1+M\frac{{\left(E-E_{0}\right)}^{2}}{\alpha^{2}}\right)\exp\left(\frac{\left(1-M\right)\ln\left(2\right)\;{\left(E-E_{0}\right)}^{2}\;}{\alpha^{2}}\right)}\;,\;E\leq E_{0} \end{array}\right.,$$

And the direct form of the step function is:

$$I_{mod}\left(E\right)=\left\{\begin{array}{c} I_{0}\;,\;E_{0}-E_{gap}<E<E_{0}\;\\ \;\\ I_{1}\;,\;E<E_{0}-E_{gap}-E_{s}\\ \;\\ I_{1}-\frac{\left(I_{1}-I_{0})({E-(E}_{0}-E_{gap}-E_{s})\right)}{E_{s}},\;E_{0}-E_{gap}-E_{s}<E<E_{0}-E_{gap}\\ I_{p}\;,\;E_{0}-\delta <E<E_{0}+\delta \\ \;\\ 0,\;E>E_{0}+\delta \end{array}\right.$$

where $E_{gap}$ is the band gap, $E_{s}$ is the width of the inclined part of the step, $E_{0}$ is the position of the elastic peak, M is the contribution of the Lorentz function to the elastic peak, $\beta^{2}$ is the width of the first half of the elastic peak, $\alpha^{2}$ is the width of the second half of the elastic peak, $E_{0}$ is the position of the elastic peak, $I_{p}$ is the intensity of the elastic peak, δ is the width of the elastic peak before the instrumental broadening (δ = 0.15 eV), $I_{1}$ is the step height, and $I_{0}$ is the signal level in the forbidden band.

**Figure 7:**
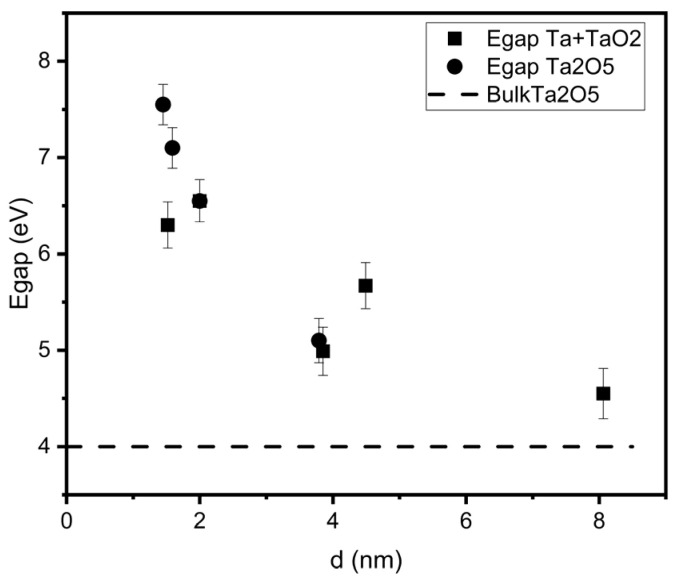
Dependence of the band gap width on the diameter of nanoclusters. Square markers indicate the band gap width for Ta+TaOx clusters, round markers indicate the band gap width for Ta_2_O_5_ clusters, and the dotted line indicates the band gap width of bulk Ta_2_O_5_.

Using this technique, the dependence of the band gap on the nanocluster diameter was obtained for both sample groups, which is shown in Figure 7.

For nanocluster diameters greater than 8 nm, the band gap of the nanocluster film becomes close to the value for bulk Ta_2_O_5_. However, when the nanocluster diameter decreases to less than 6 nm, the band gap increases significantly, reaching 7.55 eV for Ta_2_O_5_ nanoclusters and 6.55 eV for Ta+TaOx nanoclusters. For both types of nanoclusters, the band gap changes due to the quantum confinement effect at d < 6 nm. This change in band gap is much larger than that observed in [8] for nanoclusters oxidized in the atmosphere, where the band gap changed from 4.72 eV to 4.47 eV. This may be due to the different chemical composition and stoichiometry of the nanoclusters in these experiments, as well as the fact that oxidation occurred in the atmosphere, so it was impossible to guarantee the sample purity, the constant thickness of the shell, and the homogeneity of oxidation of different clusters.

According to [23], the effective mass of charge carriers in nanoclusters can be calculated from the band structure $E_{c}(k)$ as follows: ${\left(\frac{1}{m^{*}}\right)}_{ij}=\frac{1}{\hbar^{2}}{\left(\frac{\partial^{2}E_{c}}{\partial k_{i}\partial k_{j}}\right)}_{k=k_{0}}=\frac{1}{m}\delta_{ij}+\frac{2}{m^{2}}\sum_{n=c}\frac{\left\langle u_{ck_{0}}\right|p_{i}\left|u_{nk_{0}}\right\rangle \left\langle u_{ck_{0}}\right|p_{j}\left|u_{ck_{0}}\right\rangle }{E_{c}\left(k_{0}\right)-E_{n}\left(k_{0}\right)}$.

In this case, the energy levels in a nanocluster can be found as a solution to the Schrödinger equation for a particle in a spherically symmetric potential well [24,25] as follows: $E_{nl}=\frac{\hbar^{2}}{2m}\frac{\chi_{nl}^{2}}{R^{2}}$ and wave vector $k=\frac{1}{R}\chi_{nl},$ where $\chi_{nl}$ are the roots of the equation $j_{l}\left(kR\right)=0$, where $j_{l}\left(kr\right)=\sqrt{\frac{\pi }{2kr}}J_{l+\frac{1}{2}}\left(kr\right)$, R is the nanocluster radius, a is the interatomic distance, k is the wave vector, m is the electron mass, ℏ is the reduced Planck constant, m* is the effective electron mass, $\delta_{ij}$ is the Kronecker symbol, and $J_{l+\frac{1}{2}}$ is the Bessel function.

**Figure 8:**
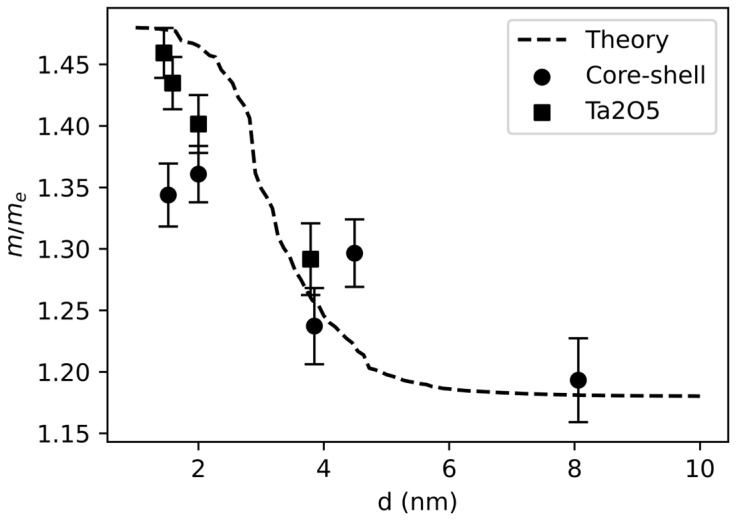
Dependences of the effective mass of electrons in core–shell tantalum nanoclusters and Ta_2_O_5_ nanoclusters on the cluster diameter.

Using the similar approximation as in [26], if we assume that the matrix element values are close to each other: $\left\langle u_{ck_{0}}\right|p\left|u_{nk_{0}}\right\rangle \approx B\hbar /a$, and the band gap of nanoclusters depends on their radius (R) as $E_{g}=\Delta E_{g}^{0}+\frac{\hbar^{2}\chi^{2}}{2mR^{2}}$, then $\frac{m}{m^{*}}\approx 1+2\frac{B\frac{\hbar^{2}}{ma^{2}}}{E_{g}}$. Where B is the model parameter (shows how many times the average momentum is larger than ℏ/a), a is the interatomic distance, m is the effective mass of an electron in the bulk material, m* is the effective mass of an electron in a nanocluster. Based on the experimental values of the band gap of nanoclusters, the dependence of the effective mass of electrons in core–shell tantalum nanoclusters and Ta_2_O_5_ nanoclusters on the nanocluster diameter was obtained using this formula. These dependences were compared with the numerical calculation, and the optimal values of the parameter B for both types of nanoclusters were found. For core–shell nanoclusters B=2.20±0.21, for Ta_2_O_5_ nanoclusters B=2.00±0.11. The values of electron effective masses for both types of nanoclusters calculated using the found parameter values and the theoretical curve found by the numerical method are shown in Figure 8.

With small sizes of nanoclusters, the effective mass of electrons increases significantly, decreasing with the increase of the diameter, and reaches a constant at diameters d > 6 nm.

## 4. Conclusions

In summary, two types of monodisperse nanocluster film samples with spherical cluster diameters in the range of 1.5–8 nm were obtained: tantalum oxide (V) nanoclusters and core–shell nanoclusters. A band gap dependence on the diameter of the nanocluster was found, which indicates the manifestation of quantum-size effects with decreasing cluster size. In the case of tantalum oxide nanoclusters, with a decrease in the nanocluster radius from 6 nm to 1.5 nm, a sharp change in the band gap is observed. The energy gap changes from values, therefore typical for bulk tantalum oxide, to values ~1.9 times greater. Core–shell nanoclusters exhibit similar behavior of this dependence, but the band gap width increases 1.57 times. In addition, the nature of the dependence becomes non-monotonic, which may be due to the discreteness of the electron levels in the metal core of the nanocluster at its size d < 2 nm and the metal–non-metal transition. The ability to modify the band gap width of the nanocluster film in a wide range might allow the electronic and optical properties of the material to be regulated, which is important for the development of selective optical coatings, nonlinear optical materials and photodetectors.

## Data Availability

Data are contained within the article.

## References

[1] Matsui T., Watanabe H., Somekawa S., Yanagida S., Oaki Y., Imai H. (2024). The Size-Dependent Valence and Conduction Band-Edge Energies of Cu Quantum Dots. Chem. Commun..

[2] Revesz A.G., Reynolds J.H., Allison J.F. (1976). Optical Properties of Tantalum Oxide Films on Silicon. J. Electrochem. Soc..

[3] Schwyn Thöny S., Bärtschi M., Batzer M., Baselgia M., Waldner S., Steinecke M., Badorreck H., Wienke A., Jupé M. (2023). Magnetron Sputter Deposition of Ta_2_O_5_-SiO_2_ Quantized Nanolaminates. Opt. Express.

[4] Borman V.D., Zenkevich A.V., Pushkin M.A., Tronin V.N., Troyan V.I. (2001). Observation of Fractal Nanoclusters on the Pulsed Laser Deposition of Gold. J. Exp. Theor. Phys. Lett..

[5] Zenkevich A.V., Pushkin M.A., Tronin V.N., Troyan V.I., Nevolin V.N., Maximov G.A., Filatov D.O., Lægsgaard E. (2002). Formation of Au Fractal Nanoclusters during Pulsed Laser Deposition on Highly Oriented Pyrolitic Graphite. Phys. Rev. B.

[6] Stefan N., Mulenko S.A., Skoryk M.A., Popov V.M., Smirnov A.B. (2023). Influence of Hybrid Fe/Cr Parameters Structures Synthesised with Laser Radiation on Their Photosensitivity. J. Mater. Sci. Mater. Electron..

[7] Lorusso A., Nassisi V., Congedo G., Lovergine N., Velardi L., Prete P. (2009). Pulsed Plasma Ion Source to Create Si Nanocrystals in SiO 2 Substrates. Appl. Surf. Sci..

[8] Borisyuk P.V., Vasilyev O.S., Lebedinskii Y.Y., Bortko D.V., Karazhanov S. (2021). Thin Ta/Ta Oxide Core-Shell Nanoparticle Film Size-Dependent Energy Structure. Mater. Lett..

[9] Vasilyev O.S., Kozlova T.I., Borisyuk P.V., Lebedinskii Y.Y. (2018). Nanocluster Size Dependence of Electronic Properties of Ta, Mo, Co, and Ni Thin Films Formed by Magnetron Sputtering. Vacuum.

[10] Vasilyev O.S., Borisyuk P.V., Lebedinskii Y.Y. (2020). The Electronic and Optical Properties of Thin Nanocluster Mo Films for Single-Photon UV Detectors. Phys. At. Nucl..

[11] Shilov V.A., Balakhnev K.M., Borisuk P.V., Bortko D.V., Vasilyev O.S. (2023). Size Dependence of the Electronic Properties of Tantalum Nanoclusters. Phys. At. Nucl..

[12] Bortko D.V., Borisyuk P.V., Shilov V.A., Vasilyev O.S., Lebedinskii Y.Y., Balakhnev K.M. (2023). Emission of Tantalum Oxide Nanocluster Thin Films at High Temperatures. Phys. At. Nucl..

[13] Kukli K., Aarik J., Aidla A., Kohana O., Uustare T., Sammelselgb W. (1995). Properties of Tantalum Oxide Thin Films Grown by Atomic Layer Deposition. Thin Solid Film..

[14] Prete P., Wolf D., Marzo F., Lovergine N. (2019). Nanoscale Spectroscopic Imaging of GaAs-AlGaAs Quantum Well Tube Nanowires: Correlating Luminescence with Nanowire Size and Inner Multishell Structure. Nanophotonics.

[15] Chaves A., Azadani J.G., Alsalman H., da Costa D.R., Frisenda R., Chaves A.J., Song S.H., Kim Y.D., He D., Zhou J. (2020). Bandgap Engineering of Two-Dimensional Semiconductor Materials. NPJ 2D Mater. Appl..

[16] Pellegrini G., Mattei G., Mazzoldi P. (2005). Finite Depth Square Well Model: Applicability and Limitations. J. Appl. Phys..

[17] Yang D.Q., Meunier M., Sacher E. (2006). Room Temperature Air Oxidation of Nanostructured Si Thin Films with Varying Porosities as Studied by X-Ray Photoelectron Spectroscopy. J. Appl. Phys..

[18] Singh V., Grammatikopoulos P., Cassidy C., Benelmekki M., Bohra M., Hawash Z., Baughman K.W., Sowwan M. (2014). Assembly of Tantalum Porous Films with Graded Oxidation Profile from Size-Selected Nanoparticles. J. Nanopart. Res..

[19] Zhang Y.X., Wang Y.H. (2017). Nonlinear Optical Properties of Metal Nanoparticles: A Review. RSC Adv..

[20] Saleem S., Jameel M.H., Yasin A., Mayzan M.Z.H.B., Ullah A., Althubeiti K., Aljohani M., Bashir J. (2024). A Band Gap and Photoluminescence Properties Engineering in BaO Semiconductor for Ultraviolet (UV) Photodetector Applications: A Comprehensive Role of Co-Doping. J. Colloid. Interface Sci..

[21] Fadley C.S., Baird R.J., Siekhaus W., Novakov T., Bergstrom S.A.L. (1974). Surface Analysis and Angular Distributions in X-Ray Photoelectron Spectroscopy. J. Electron. Spectros. Relat. Phenom..

[22] Martin J.E., Herzing A.A., Yan W., Li X.Q., Koel B.E., Kiely C.J., Zhang W.X. (2008). Determination of the Oxide Layer Thickness in Core-Shell Zerovalent Iron Nanoparticles. Langmuir.

[23] Solanki A.K., Ahuja R., Auluck S. (1992). Fermi Surface Characteristics and Enhancement Factors for Tantalum. Pramana-J. Phys..

[24] Efros A.L., Efros A.L. (1982). Interband Absorption of Light in a Semiconductor Sphere. Sov. Phys. Semicond..

[25] Baimuratov A.S., Rukhlenko I.D., Turkov V.K., Ponomareva I.O., Leonov M.Y., Perova T.S., Berwick K., Baranov A.V., Fedorov A.V. (2014). Level Anticrossing of Impurity States in Semiconductor Nanocrystals. Sci. Rep..

[26] Nikiforov V.N., Oksengendler B.L., Turaeva N.N., Marasulov M.B. (2013). Problem on the Effective Electron Mass in Nanoclusters. Russ. Phys. J..

